# Development of body, head and brain features in the Australian fat-tailed dunnart (*Sminthopsis crassicaudata*; Marsupialia: Dasyuridae); A postnatal model of forebrain formation

**DOI:** 10.1371/journal.pone.0184450

**Published:** 2017-09-07

**Authors:** Rodrigo Suárez, Annalisa Paolino, Peter Kozulin, Laura R. Fenlon, Laura R. Morcom, Robert Englebright, Patricia J. O’Hara, Peter J. Murray, Linda J. Richards

**Affiliations:** 1 The University of Queensland, Queensland Brain Institute, Brisbane, Queensland, Australia; 2 The University of Queensland, School of Agriculture & Food Science, Gatton, Queensland, Australia; 3 The University of Queensland, School of Biomedical Sciences, Brisbane, Queensland, Australia; Instituto Cajal-CSIC, SPAIN

## Abstract

Most of our understanding of forebrain development comes from research of eutherian mammals, such as rodents, primates, and carnivores. However, as the cerebral cortex forms largely prenatally, observation and manipulation of its development has required invasive and/or *ex vivo* procedures. Marsupials, on the other hand, are born at comparatively earlier stages of development and most events of forebrain formation occur once attached to the teat, thereby permitting continuous and non-invasive experimental access. Here, we take advantage of this aspect of marsupial biology to establish and characterise a resourceful laboratory model of forebrain development: the fat-tailed dunnart (*Sminthopsis crassicaudata*), a mouse-sized carnivorous Australian marsupial. We present an anatomical description of the postnatal development of the body, head and brain in dunnarts, and provide a staging system compatible with human and mouse developmental stages. As compared to eutherians, the orofacial region develops earlier in dunnarts, while forebrain development is largely protracted, extending for more than 40 days versus ca. 15 days in mice. We discuss the benefits of fat-tailed dunnarts as laboratory animals in studies of developmental biology, with an emphasis on how their accessibility in the pouch can help address new experimental questions, especially regarding mechanisms of brain development and evolution.

## Introduction

The six-layered cerebral cortex, also known as the isocortex, is a region of the forebrain only present in mammals, and participates in daily functions such as sensory integration, motor planning, attention and learning. Comparative studies of brain development across species have provided critical insights not only into the mechanisms of circuit formation, but also the conservation and change of such mechanisms during evolution. However, a critical challenge in comparative neuroscience is to determine whether and how developmental processes in one species can be compared to those of another species. Direct comparisons of development between species are often obscured by differences in the relative rates of trait formation (heterochrony). For example, the three major mammalian groups, *i*.*e*., monotremes, marsupials and eutherians, show remarkable heterochronies in face, forelimb and brain formation that likely reflect differences in pre- and postnatal development [1–11]. Therefore, to facilitate our understanding of mechanistic and evolutionary questions about mammalian development, we need to integrate the timing of developmental events in different species into a shared scale.

Previous efforts to categorise development as discrete series have helped overcome this issue by establishing comparable stages across model organisms [4, 12–15]. Here, we characterise postnatal development of the Australian marsupial fat-tailed dunnart, *Sminthopsis crassicaudata* (Marsupialia: Dasyuridae), using a standardised staging system that matches human and mouse development (i.e., the Carnegie and Thieler staging systems, respectively). Notably, while most of the neurons and connections of the cerebral cortices form before birth in eutherian species, in marsupials this occurs almost exclusively after birth and during a prolonged period of time, thus offering experimental access to early events of forebrain development *in vivo* [6, 7, 10–20]. We outline our protocols for the breeding and care of this species in captivity to facilitate its use as a laboratory model of developmental biology, and identify its major developmental milestones based on whole body, craniofacial and brain anatomy features across stages, from birth to weaning. Finally, we compare dunnart development with that of other mammals and highlight the advantages of using this species as an experimental model to study specific events of forebrain development *in vivo*.

## Materials and methods

### Animal welfare

All animal procedures, including laboratory breeding, were approved by The University of Queensland Animal Ethics Committee and the Queensland Government Department of Environment and Heritage Protection, and were performed according to the current Australian Code for the Care and Use of Animals for Scientific Purposes (NHMRC, 8^th^ edition, 2013), as well as international guidelines on animal welfare.

### Breeding colony

We established a breeding colony of fat-tailed dunnarts (*Sminthopsis crassicaudata*) at the Native Wildlife Teaching and Research Facility, The University of Queensland, from founder animals sourced from captive colonies at the University of Newcastle (NSW), Remabi Park (SA), and the University of South Australia (SA), between June 2012 and December 2013. The genetic integrity of our colony is kept via rotation of breeders using the Poiley outbreeding system [21]. Breeding boxes were set in a male:female ratio of 1:1 (virgin males) to 1:3 (experienced males) per cage, with cage dimensions of 520 x 335 x 95 (mm). Female dunnarts reach sexual maturity at approximately 5 months (average age of females’ first-litter is 210 days), and the first females to conceive per litter were kept as new breeders to select for fertility. Weaned and adult dunnarts not included in mating groups were housed individually in cages containing 10 mm corn cob substrate, shredded newspaper, nest boxes (cardboard tubes and boxes), a sand bowl, a water bowl, a 7–12 cm rock, a food bowl, and a drinking bottle. Animals were fed a daily diet consisting of cat biscuits (Adult Cat Total Wellbeing, chicken, Advance) *ad libitum*, beef mince-mix (w/w, 78% lean beef mince, 5% ground cat kibble, 14% Wombaroo small carnivore food, and 3% of balanced calcium powder; 0.5g for weaners/non-breeders, and 4g lactating females), and live mealworms (*Tenebrio molitor* larva; 3 for weaners/non-breeders, and 6–10 for lactating females). Additionally, once per week, animals received 5-10g sunflower seeds, 5-10g hard-boiled egg and 5-10g apple. The room was maintained on a 16:8h light:dark cycle, humidity between 30 and 60% and temperature between 22 and 26°C. To optimise breeding success, false winters were set, usually around June and/or December, by gradually reversing the light cycle and dropping temperature by 2–3°C for 2–4 weeks.

To check for joeys’ presence, females in breeding cages were gently retrieved from a bottomless hiding box with one hand, allowing gentle inspection of the pouch with the other hand. In non-parous females, the pouch is usually tight and full of pale and dry hair. On the other hand, the pouch of pregnant and oestrous females is easier to open and hairless [22]. Females with pouch-young can be detected by a moist pouch and presence of joeys attached to the teats. Teats not used by joeys are small and opaque, while the ones with attached young are prominent and highly vascularised.

### Anaesthesia and tissue collection

For temporary sedation, adult female dunnarts with joeys were transferred into a gas anaesthesia induction chamber with isoflurane 5%, delivered in oxygen at a flow rate of 200 mL/Kg/min, followed by isoflurane 2–5% supplied through a rubber mask throughout the procedure (ZDS MINI Qube Anaesthetic System). This allows careful examination of joeys in a non-invasive manner. Joeys were removed from the teat by gently pulling with forceps and euthanised with an intraperitoneal injection of 0.05–0.5 mL solution of sodium pentobarbitone (1/50 v/v Lethabarb, Virbac, corresponding to 190 mg Lethabarb per kg body weight), or 5–15 min ice anaesthesia for joeys younger than postnatal day (P) 45, followed by transcardial perfusion of 0.9% NaCl and 4% paraformaldehyde (PFA), or immersion-fixation in 4% PFA for joeys younger than P15.

### Histology and microscopy

Following perfusion, joeys younger than 2 weeks of age underwent paraffin embedding and had their heads sectioned in the coronal plane at 12 μm in a sliding microtome (Leica SM 2000R). Sections were mounted, dewaxed and stained with haematoxylin and eosin. Older joeys had their brains dissected, photographed (Lumix DMC-LX7, Panasonic), embedded in 3.4% agarose blocks and sectioned at 50 μm using a vibratome (Leica VT1000S). These sections were stained for 10 minutes with 0.1% DAPI (Invitrogen), and then washed and coverslipped with ProLong Gold (Invitrogen). Brightfield and fluorescence microphotographs were obtained using a Zeiss upright Axio-Imager microscope fitted with Axio-Cam HRc and HRm cameras, and captured using AxioVision software (Carl Zeiss). Photoshop and Illustrator (Adobe) were used to adjust levels, contrast and prepare figure panels.

### Morphometry and statistics

To track joey morphometry, pouches were exposed as described above and the joeys were photographed (Lumix DMC-LX7, Panasonic) at regular intervals until P45. Crown-rump length, skull width (bi-parietal diameter), and forelimb and hindlimb length were measured from the pictures that included identical calibration tools (Fiji, NIH). Details about the appearance of the joeys were noted across stages, paying particular attention to fur, mouth, eyes, ears, nose, skin, forelimbs, fingers, hindlimbs, and toes. Growth curves and 95% confidence intervals were generated using non-linear semilog fitting of morphometric measurements following the least-squares method. Goodness-of-fit was quantified as R squared (R^2^) with 95% confidence intervals and degrees of freedom (d.f.) > 12. Normality tests of residuals were passed in all cases (D’Agostino-Pearson omnibus K2; Prism 7, GraphPad). Measurements are presented as mean ± standard error of the mean.

## Results

### Developmental morphometry and growth curves of postnatal *S*. *crassicaudata*

**Fig 1:**
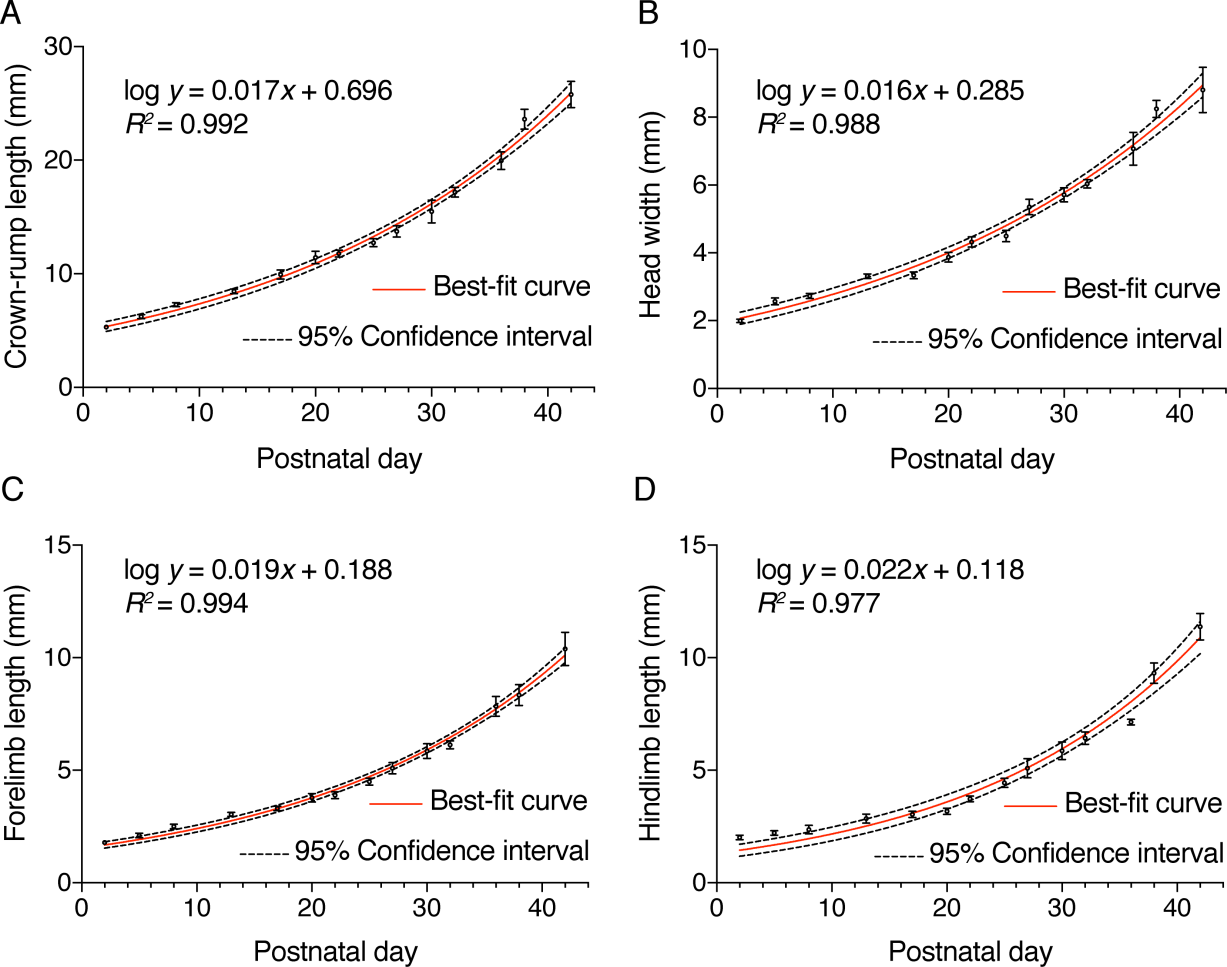
Developmental growth curves of postnatal *S*. *crassicaudata*. Semilogarithmic relationships between the postnatal age (in days) and crown-rump length (A), head width (B), forelimb length (C), and hindlimb length (D). Mean ± standard error of the mean, as well as best-fit regression curves (red lines) and 95% confidence intervals (dotted lines) are indicated in each case. Non-linear functions whereby *y* indicates the corresponding measurements (in mm) and *x* the age in days, as well as *R*^*2*^ values are indicated. See Tables 1 and 2 for additional information.

To characterise growth and developmental morphometry of postnatal *S*. *crassicaudata*, we measured crown-rump length, maximum head width, forelimb length and hindlimb length from at least four different individuals (median 9, mean 14.3) per measurement and age range (see Table 1). Semi-logarithmic relationships between each of these measurements and ages were determined, including best-fit regression curves and 95% confidence intervals (Fig 1). Crown-rump length, skull width and forelimb length showed good non-linear fit (R^2^ ≥ 0.985; Table 2), indicating that morphometry alone can be used to infer developmental age in the case of incomplete data, such as for example from field measurements where date of birth is unknown [22]. Interestingly, however, body size is often more similar between littermates than to age-matched joeys of different sized litters, suggesting that differences in the metabolic/nutritional state of the mothers and/or the number of teats/joeys per litter could be sources of variability [7, 9].

**Table 1:** Morphometric measurements in postnatal *S*. *crassicaudata*.

Age (days)	Crown-rump length (mm)	Skull width (mm)	Forelimb length (mm)	Hindlimb length (mm)
P2/4	5.32 ± 0.11 *(n = 59)*	1.99 ± 0.05 *(n = 32)*	1.79 ± 0.04 *(n = 58)*	2.01 ± 0.11 *(n = 4)*
P5/7	6.24 ± 0.18 *(n = 19)*	2.57 ± 0.11 *(n = 14)*	2.11 ± 0.10 *(n = 15)*	2.22 ± 0.11 *(n = 4)*
P8/12	7.30 ± 0.20 *(n = 34)*	2.73 ± 0.08 *(n = 15)*	2.51 ± 0.10 *(n = 34)*	2.37 ± 0.18 *(n = 9)*
P13/16	8.46 ± 0.20 *(n = 35)*	3.31 ± 0.07 *(n = 29)*	3.05 ± 0.09 *(n = 34)*	2.85 ± 0.20 *(n = 7)*
P17/19	9.93 ± 0.40 *(n = 14)*	3.34 ± 0.10 *(n = 7)*	3.31 ± 0.10 *(n = 15)*	3.04 ± 0.15 *(n = 9)*
P20/21	11.43 ± 0.56 *(n = 9)*	3.87 ± 0.14 *(n = 8)*	3.79 ± 0.19 *(n = 10)*	3.18 ± 0.15 *(n = 7)*
P22/24	11.80 ± 0.30 *(n = 16)*	4.32 ± 0.15 *(n = 12)*	3.89 ± 0.15 *(n = 16)*	3.74 ± 0.13 *(n = 15)*
P25/26	12.74 ± 0.35 *(n = 18)*	4.50 ± 0.17 *(n = 11)*	4.49 ± 0.15 *(n = 19)*	4.45 ± 0.20 *(n = 13)*
P27/29	13.73 ± 0.51 *(n = 6)*	5.35 ± 0.22 *(n = 7)*	5.09 ± 0.26 *(n = 7)*	5.10 ± 0.43 *(n = 6)*
P30/31	15.50 ± 1.01 *(n = 7)*	5.71 ± 0.21 *(n = 6)*	5.86 ± 0.33 *(n = 7)*	5.87 ± 0.40 *(n = 7)*
P32/35	17.20 ± 0.45 *(n = 23)*	6.04 ± 0.12 *(n = 13)*	6.13 ± 0.18 *(n = 23)*	6.43 ± 0.27 *(n = 20)*
P36/37	19.95 ± 0.79 *(n = 5)*	7.07 ± 0.49 *(n = 4)*	7.84 ± 0.44 *(n = 4)*	7.14 ± 0.13 *(n = 4)*
P38/40	23.62 ± 0.89 *(n = 7)*	8.24 ± 0.26 *(n = 9)*	8.35 ± 0.46 *(n = 9)*	9.32 ± 0.45 *(n = 8)*
P42/45	25.80 ± 1.17 *(n = 4)*	8.80 ± 0.67 *(n = 4)*	10.40 ± 0.74 *(n = 4)*	11.38 ± 0.58 *(n = 4)*

Values indicate mean ± standard error of the mean and sample sizes.

**Table 2:** Non-linear regression statistics for *S*. *crassicaudata* growth curves.

Semilog line—X is linear, Y is log		Crown-rump length (mm)	Head width (mm)	Forelimb length (mm)	Hindlimb length (mm)
Best-fit values					
	Y-intercept	0.6959	0.285	0.188	0.1182
	Slope	0.01709	0.01587	0.01945	0.02188
Std. Error					
	Y-intercept	0.01695	0.01822	0.01753	0.03867
	Slope	0.0005084	0.0005532	0.000515	0.001114
95% CI (profile likelihood)					
	Y-intercept	0.6579 to 0.7323	0.2442 to 0.324	0.1483 to 0.2261	0.0267 to 0.2019
	Slope	0.01599 to 0.01822	0.01468 to 0.0171	0.01832 to 0.02061	0.01943 to 0.02449
Goodness of fit					
	Degrees of Freedom	12	12	12	12
	R squared	0.9916	0.9883	0.9936	0.9769
	Absolute Sum of Squares	4.32	0.681	0.5457	2.4
	Sy.x	0.6	0.2382	0.2132	0.4472
Normality of residuals					
	D'Agostino & Pearson omnibus K2	5.533	1.883	0.9898	1.56
	P value	0.0629	0.3901	0.6096	0.4583
	Passed normality test (alpha = 0.05)?	Yes	Yes	Yes	Yes

### A postnatal staging system for *S*. *crassicaudata*

**Fig 2:**
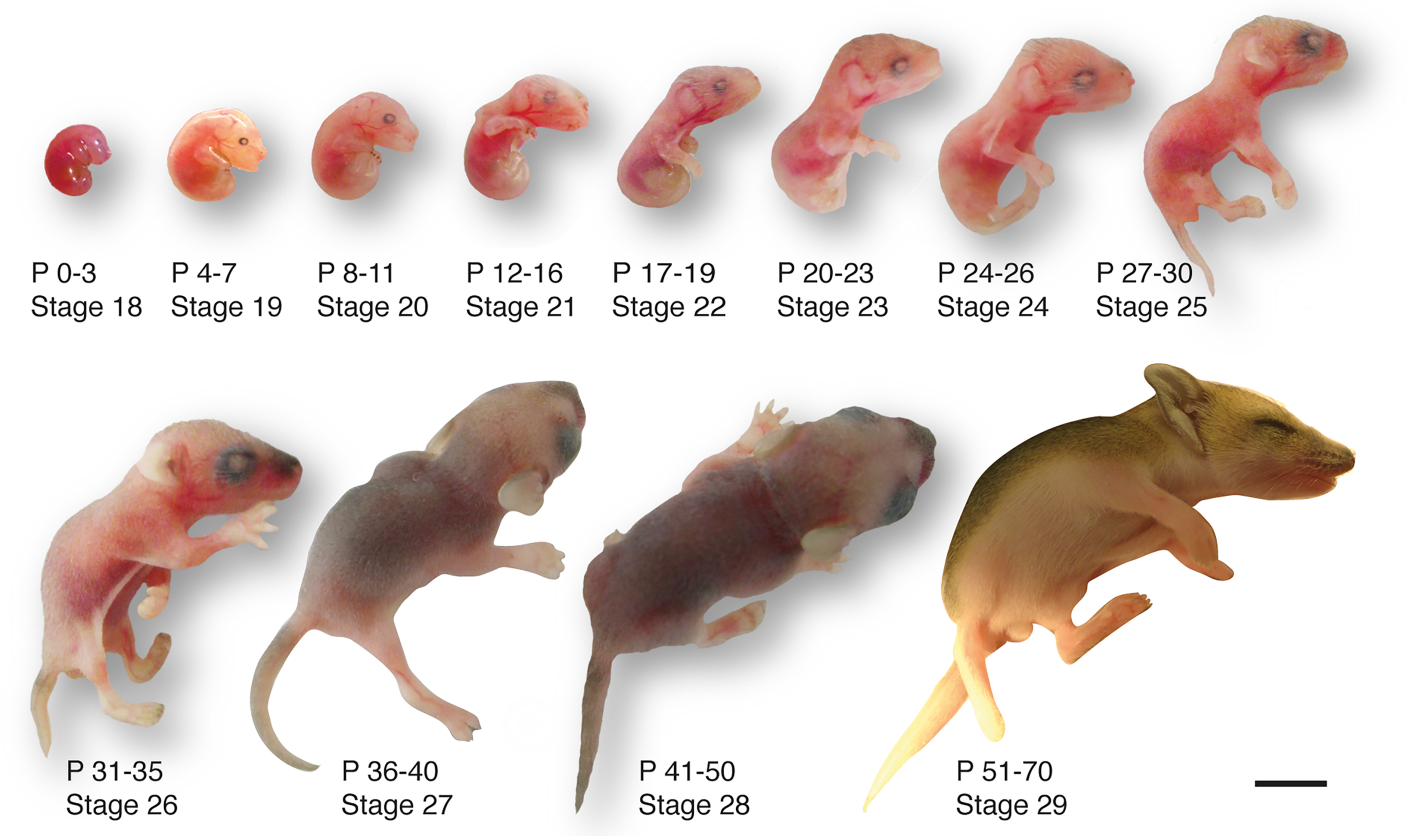
Developmental series of postnatal *S*. *crassicaudata*. A system of twenty-nine arbitrary stages from conception to weaning is included to match equivalent stages in human (Carnegie staging system) and mouse (Thieler staging system). Birth occurs at stage 18. Corresponding age of each stage is included as a range of postnatal days (P). See Table 3 for further details. Scale bar: 5 mm.

To provide a developmental staging system that would account for inter-individual size variability, while allowing inter-species comparisons, we categorised dunnart development within both the Carnegie staging system for human development [13, 14], and the Thieler staging system for mouse development [15]. We considered formation of the eye as a comparative “anchor point”, as its developmental timing is highly conserved from birds to mammals [23]. The Carnegie human system comprises 23 stages from conception to the formation of most adult structures (ca. 60 days/8-9 weeks), after which the embryo is considered a foetus. The Thieler mouse system is based on milestones that match the Carnegie stages, and includes five additional stages that end postnatally with eye opening. For dunnarts, we have developed a comparable system, whereby embryos are born at equivalent stage 18 and leave the pouch at stage 29, ca. 70 days later (Fig 2). Intrauterine gestation lasts for 13–14 days [24], after which multiple 3–5 mm newborns must reach the pouch and attach to one of the 8–10 teats to complete development. One mother out of 105 in our study had 11 teats and 11 joeys, prompting the speculation that more newborns than teats available might be born and, therefore, that teat number is a limiting factor for litter size. Mothers in our study had an average of six and a mode of seven joeys.

### Early postnatal anatomy of newborn *S*. *crassicaudata* (stage 18)

**Fig 3:**
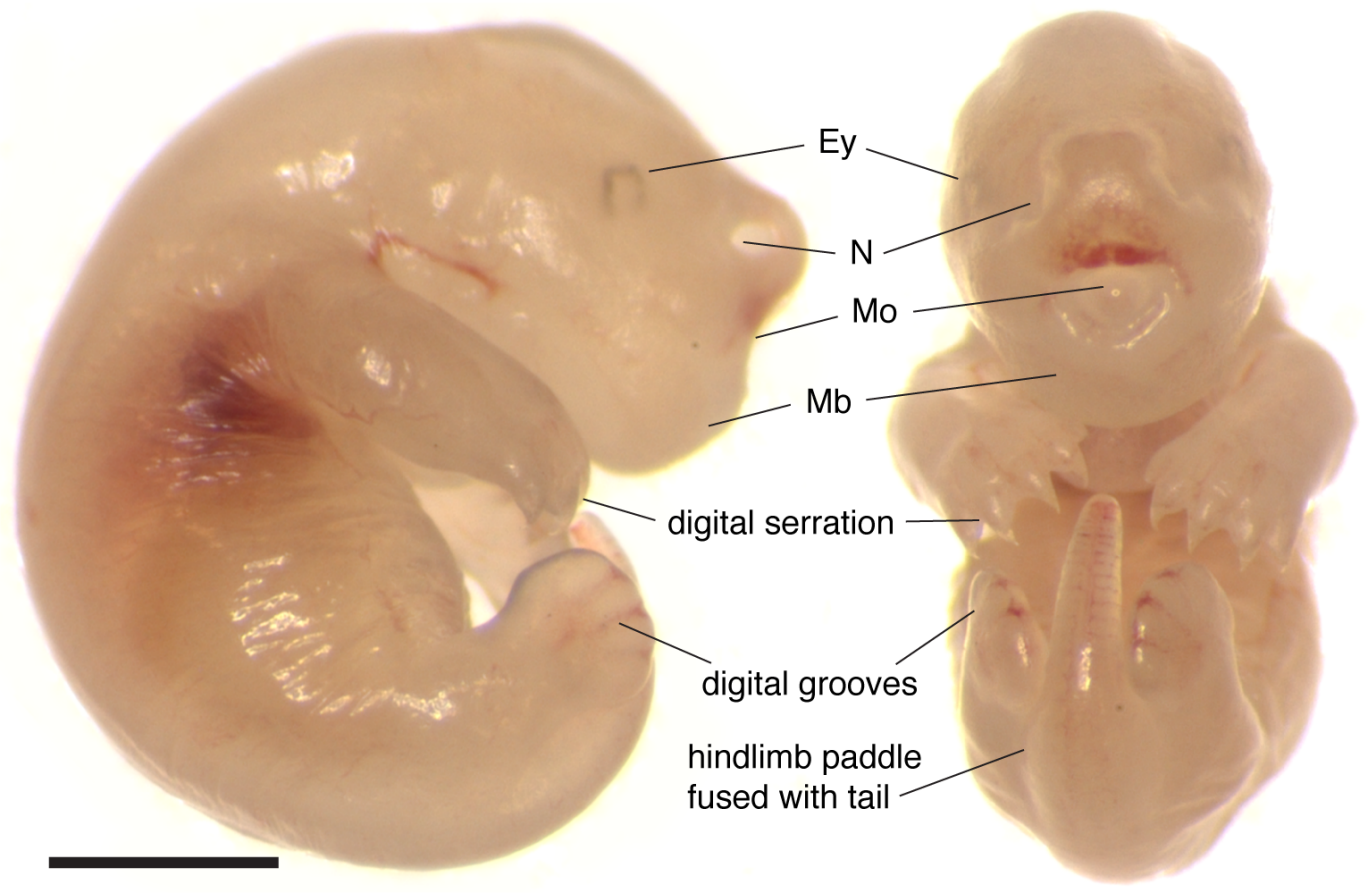
External body features of newborn *S. crassicaudata*. Microphotograph of a stage 18 newborn dunnart as seen from the right and from the front. Note the prominent orofacial features, including fused mandible (Mb), and wide nostrils (N). The eyes (Ey) are small and lightly pigmented. Forelimbs are more advanced than hindlimbs, with digits serrated as compared to completely fused. Scale bar: 1mm.

**Fig 4:**
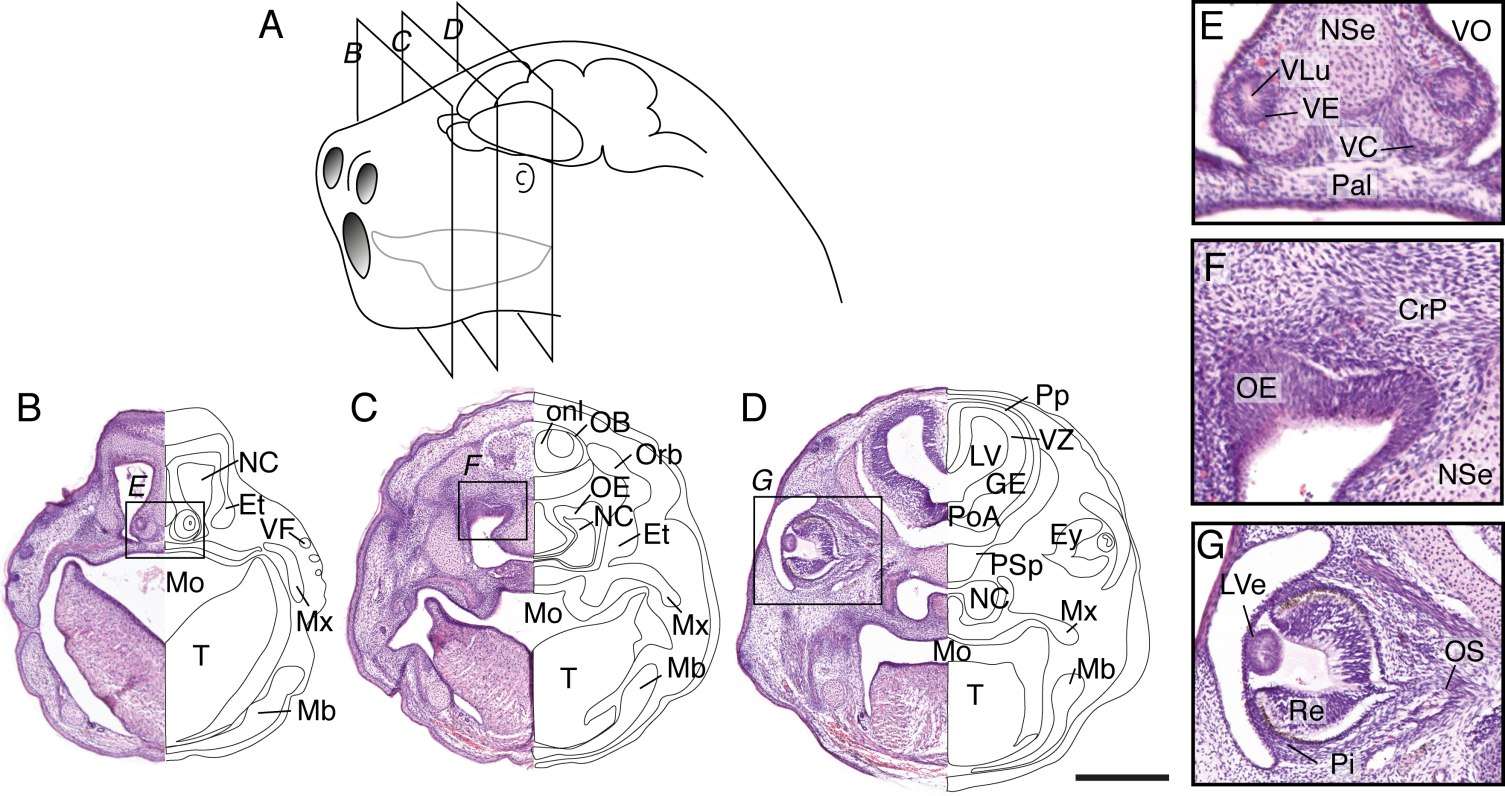
Craniofacial features of newborn *S*. *crassicaudata*. (A) Schematic of a stage 18 dunnart head (postnatal day 0–3) indicating the relative size and position of nostrils, mouth, tongue, eyes and brain, as well as the section planes shown in (B-D). (B-D) Haematoxylin/eosin staining of a rostral-caudal series through the planes depicted in panel A reveals orofacial, sensory and brain structures of newborn dunnarts, including a prominent tongue (T) that encloses the teat inside the mouth (Mo) reaching the lateral margins of the palate (Pal). Large nasal cavities (NC) with fused palate (Pal) and nasal septum (NSe) can be seen, including condensation of mandibular (Mb) and maxillary (Mx) processes, eyes (Ey) fully covered by skin, and scattered vibrissal follicles (VF) in the snout. Telencephalic structures include the olfactory bulb (OB), with surrounding olfactory nerve layer (onl), preplate (Pp), ventricular zone (VZ) and ganglionic eminences (GE) as well as a large lateral ventricle (LV). E-F, inset of the regions highlighted in B-D depicting developing sensory structures. E, the vomeronasal organ (VO) includes a lumen (VLu), neuroepithelium (VE) and capsule (VC) at the base of NSe and Pal. F, a thick olfactory neuroepithelium (OE) is located at the roof of the NC, immediately below the presumptive cribiform plate (CrP). G, eye development has just undergone closure of the lens vesicle (LVe), the pigment epithelium (Pi) outlines a dark ring around cells of the retina (Re). Et, ethmoid bone; Orb, orbital bone; PoA, preoptic area; PSp; presphenoid bone. Scale bar: 500 μm.

Newborn dunnarts have translucent and glossy skin. The forelimbs display alternating movements and are larger and more developed than the hindlimbs. The forelimbs have a distinguishable elbow joint and hand digits are partially fused (digital serration), while the forelimbs are proximally fused to the tail, mostly not mobile, and the feet digits are completely fused (paddle shape), although digital grooves are noticeable (Fig 3 and Table 3). The small eyes have a faint but noticeable ring of pigment. While eutherian mammals at equivalent stages have not yet undergone formation of the mouth apparatus, newborn dunnarts have a fully fused palate and well developed tongue (Figs 3 and 4). The lateral margins of the tongue press tightly around the teat and against the palate to allow active milk suckling. The presence of an olfactory and vomeronasal neuroepithelium (Fig 4E and 4F) in newborns raises the question of whether these systems are functional at birth. The olfactory bulb receives sensory axons traversing the cribiform plate, and the telencephalic vesicles include well-developed ganglionic eminences, an olfactory (piriform) cortex and a thin dorsal pallium (presumptive isocortex) that consists of a proliferative ventricular zone and preplate (Fig 4D). Eye development at birth has just undergone closure of the lens vesicle but no lens fibres are present as yet (Fig 4G).

**Table 3:** Developmental features across postnatal stages of *S*. *crassicaudata*.

Stage	Age (postnatal days)	Body	Head	Brain
**18**	0–3	• Hairless, translucent and shiny skin. • Forelimbs with elbow. Mostly fused digits (digital serration). • Hindlimb paddle, partly fused with the tail. Completely fused digits (digital grooves). • Joeys are curled and difficult to stretch. • Milk spot visible. • Little body movement.	• Prominent mandibular prognathism (underbite). • Frontal mouth opening. • Eye pigment slightly visible. • Lens vesicle just closed. • No ear buds. • Large nostrils.	• Telencephalic vesicles present. • Dorsal pallium at stage of preplate and ventricular zone. • Large diencephalon and midbrain.
**19**	4–7	• Distinct hands. Digits partly fused. • Hindlimbs and tail buds distinguishable, still fused. • Joeys easier to uncurl. • Alternate forelimb movement.	• Darker eye pigment. • No face pigmentation. • Developing lens fibres. • Short and thin hair, only on top of the head.	• Piriform cortex and olfactory tubercle better defined.
**20**	8–11	• More opaque skin. • Small claws in separated forelimb digits. • Hindlimb digits mostly fused (digital serration). • Jugular and carotid processes defined.	• Small ear buds. • Slight pigment on top of snout. • Lens epithelium and fibres enlarged. Retinal layers slightly visible. Optic stalk.	• Prominent lateral olfactory tract, endopiriform and piriform regions. • Dorsal pallium without a defined cortical plate.
**21**	12–15	• Opaque skin. • Hindlimbs and tail unfused and well defined. • Hindlimb digits partly fused. • Joeys can be completely uncurled. • Movement of front-temporal head muscles.	• Underbite less prominent. • Mild pigment on top of snout. • Short, thick hair only on top of the head. • Thicker cornea, lens well developed. Clear retinal layers. Optic nerve.	• Internal, and external capsules clearly defined. • Anterior commissure present. • Cortical plate appears. • Larger subpallium region.
**22**	16–19	• Large, pigmented forelimb claws. • Hindlimb digits separated, with small claws. • Large jugular and carotid processes visible.	• Larger ear buds. • Darker hair on top of the head. • Darker pigment on top of snout.	• Olfactory tubercle and layer 2 of piriform cortex clearly defined.
**23**	20–23	• Short, light hair on face and body. • - Hindlimb digits well defined, with small claws.	• Underbite less prominent. • Larger, darker eye pigment. • Larger ear buds.	• Expansion of subventricular and intermediate zones. • Ventralisation of the piriform cortex.
**24**	24–26	• Short, light hair on face and body. • Darker skin, jugular and carotid processes less visible. • Longer, motile hindlimbs, with clear claws. • Overall body movement increased and responsive.	• Mandibulary and maxillary processes aligned. • Long, thick, dark hair on top of head. • Ear flap is distinguishable. • Small whiskers.	• Hippocampal commissure first visible. • Indentation of piriform and olfactory tubercle cell layers along the lateral olfactory tract. • Superior and inferior colliculi distinguishable.
**25**	27–30	• Slightly darker body hair. • Legs and tail very well developed. Hindlimb claws slightly pigmented. • Less curved and more motile body.	• Snout progression (slight maxillary overbite). • Mouth still fused. • Larger and darker eye pigment. • Ear lobes better defined.	• Indentation of the rhinal fissure. • Ventral expansion of the developing cortical plate.
**26**	31–35	• Longer, darker hair on face and body, especially on tail and back. • Large digits with dark and well developed claws. • Jugular and carotid processes less visible.	• Mouth lining visible but still fused. • Eyelid grooves become apparent. • Dark, larger skin pigment patch on top of snout. Larger whiskers.	• Reduction of the subventricular zone. • Cortical layers 5 and 6 are present.
**27**	36–40	• Longer, darker hair all over body. • Jugular and carotid processes not visible. • Testes/pouch clearly visible. • Fully dependent on their mother, have never left the pouch.	• Mouth almost unfused, deep groove. • Eyelid margin is clearly distinct. • Ear canal better defined.	• Cortical layers 2–4 are defined.
**28**	41–50	• Back completely covered with dark and thick hair, abdomen hair lighter colour. • Pups, can detach and switch nipples, as well as leave the pouch temporarily. • Narrower snout.	• Mouth unfused, can be fully opened. • Very pigmented and large, but unopen, eyes. • Fully formed ears with clearly open canal.	• Further indentation of the rhinal fissure. • Further ventral progression of the cortex. • Diencephalon is fully covered by the dorsal cortex.
**29**	51–70	• Mature-looking fur in head and body. • Mature-looking narrow snout. • Pups often leave the pouch and try solid food.	• Mouth fully open. • Mature-looking whisker pads. • Eyes begin to open.	• Well defined cortical areas and regions, resembling the adult.

### Craniofacial and brain development in stages 19–21 of *S*. *crassicaudata*

**Fig 5:**
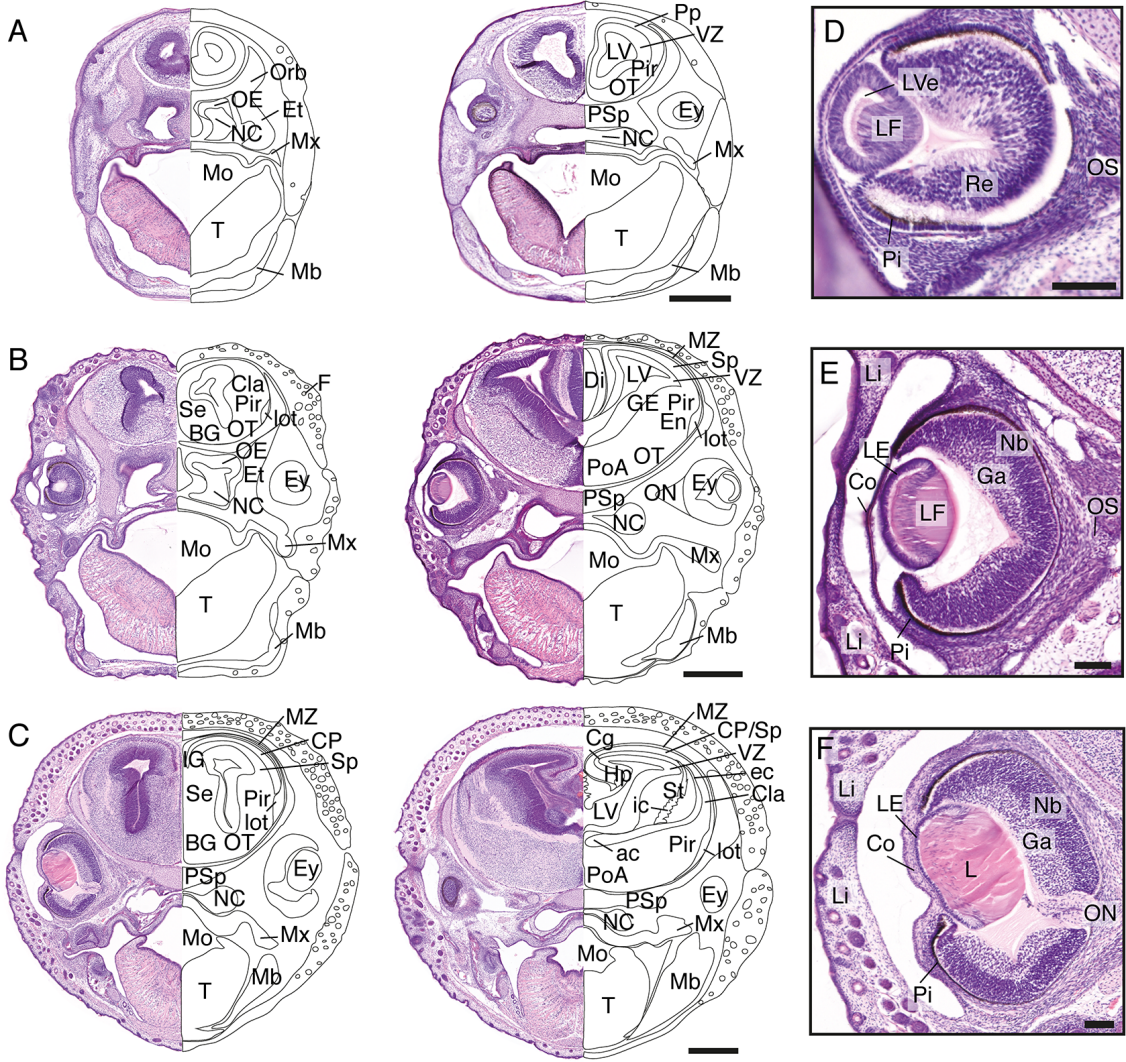
Craniofacial features of stages 19–21 *S*. *crassicaudata*. Haematoxylin/eosin staining through rostral (left) and caudal (right) regions of the head of *S*. *crassicaudata* at stages 19 (A), 20 (B), and 21 (C) in the coronal plane (postnatal days 4–7, 8–11, and 12–15, respectively). Insets of the developing eye at corresponding ages (D-F, respectively). ac, anterior commissure; BG, basal ganglia; Cg, cingulate cortex; Cla, claustrum; Co, cornea; CP, cortical plate; ec, external capsule; En, endopiriform nucleus; Ey, eye; Et, ethmoid bone; F, follicle; Ga, ganglion layer; GE, ganglionic eminence; Hp, hippocampus; ic, internal capsule; IG, indusium griseum; L, lens; LE, lens epithelium; LF, lens fibres; Li, eyelid; lot, lateral olfactory tract; LV, lateral ventricle; LVe, lens vesicle; Mb, mandibulary process; Mo, mouth; Mx, maxillary process; MZ, marginal zone; NC, nasal cavity; Nb, neuroblastic layer; OE, olfactory neuroepithelium; ON, optic nerve; Orb, orbital bone; OS, optic stalk; OT, olfactory tubercle; Pi, pigment epithelium; Pir, piriform cortex; PoA, preoptic area; Pp, preplate; PSp; presphenoid bone; Re, retinal layer; Se, septum; Sp, subplate; St, striatum; SVZ, subventricular zone; T, tongue; VZ, ventricular zone. Scale bars: 500 μm (A-C), 100 μm (E-F).

Head features of subsequent stages 19, 20, 21 of *S*. *crassicaudata* (postnatal days 4–7, 8–11, and 12–15, respectively) are presented in Fig 5A–5C. Hair follicles can be seen increasing in abundance between these stages (Figs 4 and 5), and subsequent development of thick hair over the parietal scalp remains a salient feature of early-to-mid postnatal development. Telencephalic development includes growth of the piriform cortex and expansion of the lateral olfactory tract, which becomes visible by stage 20 (Fig 5B). The ganglionic eminences are apparent by stage 20 and become striated at stage 21 by axons forming the internal capsule (Fig 5C). The isocortical preplate has split to form the marginal zone and the subplate by stage 20, followed by the emergence of a thin cortical plate by stage 21. The anterior commissure, carrying olfactory, piriform and temporal isocortical axons appears at stage 21. Eye development shows a similar developmental sequence to stage-matched mice and humans (Fig 5D–5F). Stage 19 includes formation of lens fibres, by stage 20 a thin lens epithelium, enlarged lens fibres, and neuroblastic and ganglion cell layers in the retina are apparent, and stage 21 is characterised by a mature-looking lens, a thicker cornea and a distinct optic nerve. Accordingly, pioneering axons of the dunnart optic nerve have been reported to begin growth, cross the midline, and reach their central targets, respectively, during these three stages [25, 26].

### Mid and late pouch development: Cortical layering and body changes in stages 22–29 of *S*. *crassicaudata*

Subsequent stages of *S*. *crassicaudata* comprise a progressive succession of general body features and brain development. Between stages 22–25, dunnarts undergo changes in facial morphology, with mandibular and maxillar processes progressing from a marked prognathism (underbite), to alignment and progression of the snout (Fig 2 and Table 3). Digit development begins in the forelimbs, and is not evident in the hindlimbs until stage 22.

**Fig 6:**
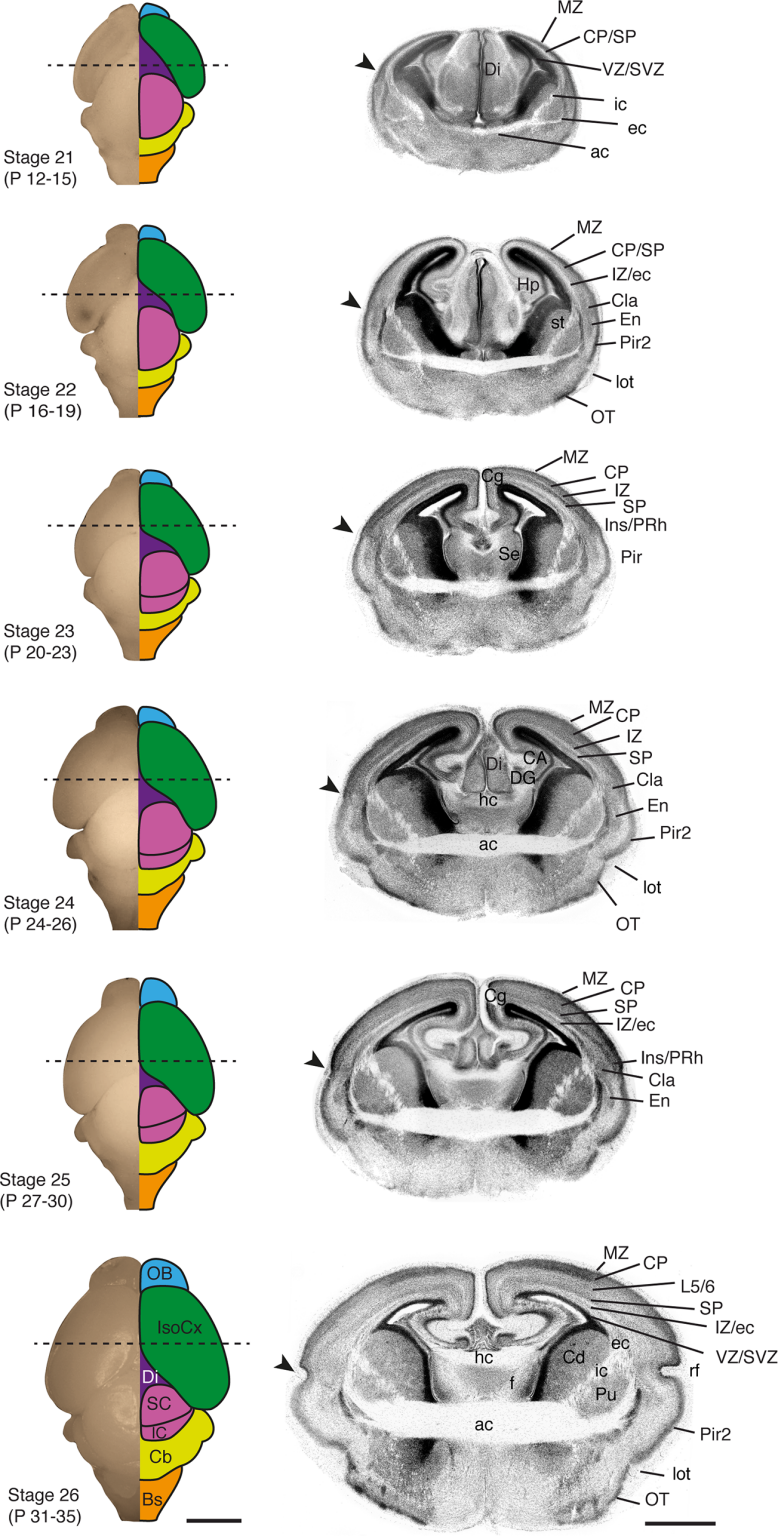
Brain features of *S*. *crassicaudata* between stages 21 and 26. Developmental series of fixed dunnart brains showing a dorsal view of the whole brain (left column) and corresponding DAPI-stained coronal section (right column) through the approximate region indicated by dotted lines. The main brain regions are colour-coded, and stages and approximate postnatal (P) days are indicated. Arrowheads show the prospective rhinal fissure (rf). ac, anterior commissure; BS, brainstem; CA, cornu ammonis hippocampi; Cb, cerebellum; Cd, caudate; Cg, cingulate cortex; Cla, claustrum; CP, cortical plate; DG, dentate gyrus; Di, diencephalon; ec, external capsule; En, endopiriform; f, fornix; hc, hippocampal commissure; Hp, hippocampus; ic, internal capsule; IC, inferior colliculus; Ins, insular cortex; IsoCx, isocortex; IZ, intermediate zone; L5/6, isocortical layers 5/6; lot, lateral olfactory tract; MZ, marginal zone; OB, olfactory bulb; OT, olfactory tubercle; Pir, piriform cortex; Pir2, piriform cortex, layer 2; PRh, perirhinal; Pu, putamen; rf, rhinal fissure; SC, superior colliculus; Se, septum; SP, subplate; SVZ, subventricular zone; VZ, ventricular zone. Scale bars: 2000 μm in whole brains, and 200 μm in coronal sections.

Principal neuron cell layers of the piriform cortex and olfactory tubercle are evident by stage 22, and become angled by the enlargement of the lateral olfactory tract (Fig 6). This is followed by the expansion of the cortical plate and underlying subplate from latero-ventral to medio-dorsal regions. In fact, the prospective claustrum and endopiriform nuclei, which arise from lateral and ventral pallial derivatives [27, 28], can be distinguished from stage 22 onwards, before the respective enlargement of the overlaying insular/perirhinal and piriform cortices, and formation of the rhinal fissure by stage 25 (Fig 6, arrowheads). In eutherians, most interhemispheric projections from the isocortex and cingulate cortex cross the midline through the corpus callosum, while in non-eutherians these projections course through the anterior commissure. In dunnarts, as in other marsupials, the anterior commissure forms at least one week before the hippocampal commissure, while in eutherians they arise closer in time, with the corpus callosum being the last telencephalic commissure to form [6, 19, 29–31]. Interestingly, dunnarts also lack the astrogliogenic remodelling of the septal midline, a process required for callosal formation in eutherians [32, 33] that could relate to heterochronies in the formation of interhemispheric circuits in the brain of mammals.

**Fig 7:**
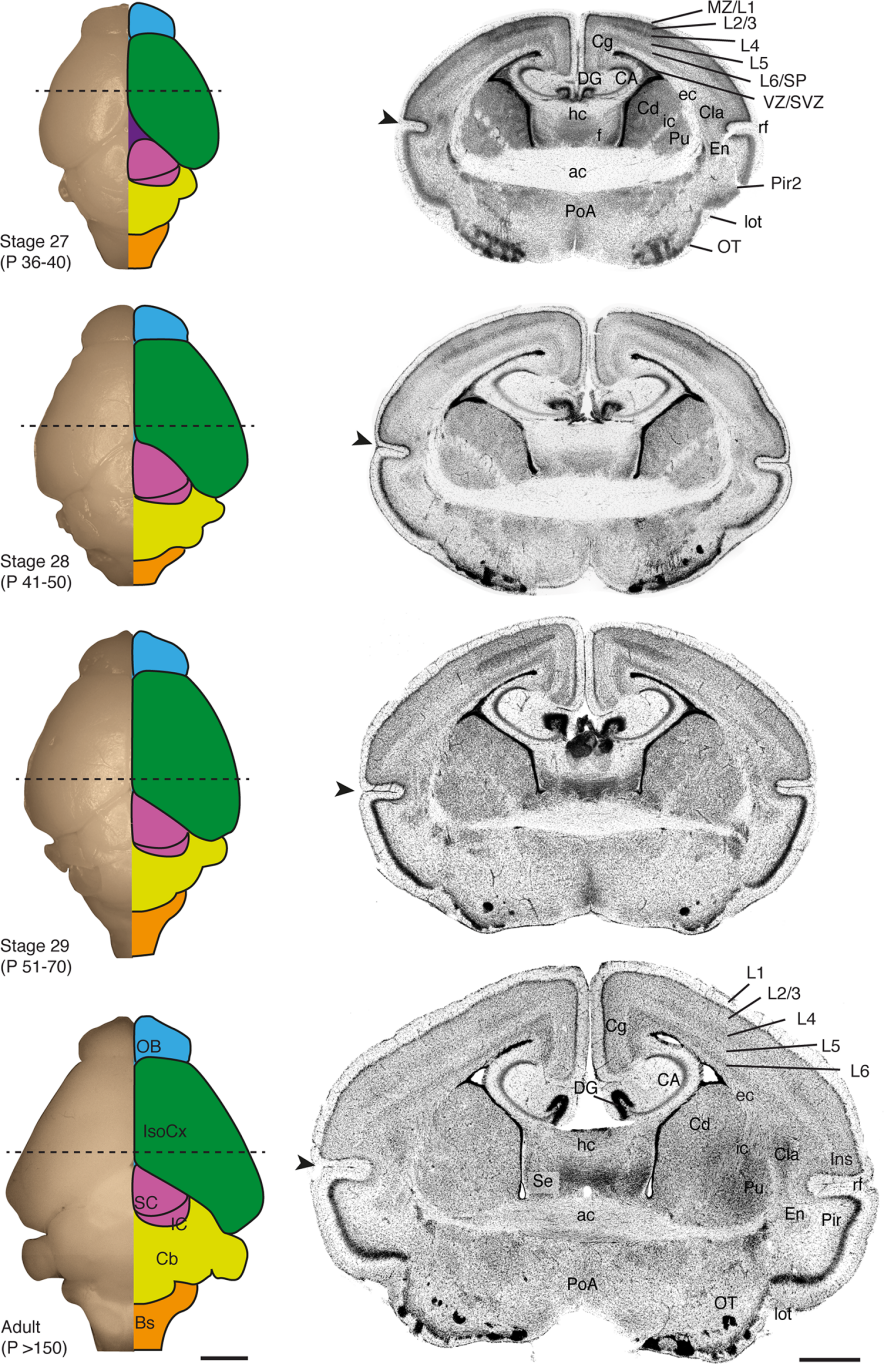
Brain features of *S*. *crassicaudata* between stages 27 and adulthood. Developmental series of fixed dunnart brains showing a dorsal view of the whole brain (left column) and corresponding DAPI-stained coronal section (right column) through the approximate region indicated by dotted lines. The main brain regions are colour-coded, and stages and approximate postnatal (P) days are indicated. Arrowheads show the rhinal fissure (rf). ac, anterior commissure; BS, brainstem; CA, cornu ammonis hippocampi; Cb, cerebellum; Cd, caudate; Cg, cingulate cortex; Cla, claustrum; DG, dentate gyrus; ec, external capsule; En, endopiriform; f, fornix; hc, hippocampal commissure; ic, internal capsule; IC, inferior colliculus; Ins, insular cortex; IsoCx, isocortex; L1-6, isocortical layers 1–6; lot, lateral olfactory tract; MZ, marginal zone; OB, olfactory bulb; OT, olfactory tubercle; Pir, piriform cortex; Pir2, piriform cortex, layer 2; PoA, preoptic area; Pu, putamen; SC, superior colliculus; Se, septum; SP, subplate; SVZ, subventricular zone; VZ, ventricular zone. Scale bars: 2000 μm in whole brains, and 200 μm in coronal sections.

A dorsal view of the brain reveals the progressive growth of the isocortical mantle (Figs 6 and 7, green schematics), which does not fully cover the dorsal diencephalon until stage 28. Development of the isocortex is largely protracted in this species, as in other marsupials [7, 8, 10], and isocortical layers do not become apparent until stage 27 (Fig 7). The piriform/insular cortex boundary (arrowheads in Figs 6 and 7) reveals the ventral expansion of the isocortex, as the rhinal fissure is positioned more ventrally between stages 24–27. Infragranular neurons that give rise to layers 5/6 of the isocortex, as well as subplate neurons, are recognisable superficial to the intermediate zone by stage 26, once supragranular neurons have begun their radial migration towards the cortical plate. The thalamo-recipient cell dense layer 4 as well as upper layers 2/3 become evident by stage 27 onwards (Fig 7). By this time, sensory axons from the thalamus accumulate in layer 4, forming modality-specific sensory areas that subsequently expand tangentially, further displacing the rhinal fissure ventrally (Fig 7).

Pre- and peri-weaning stages are characterised by the formation of the mouth lining from a deep groove, noticeable from stage 26, which allows full opening of the mouth for the first time around stage 28, once isocortical neurons are established in their final layers. After this time, joeys can be observed swapping teats and leaving the pouch temporarily. Fur begins covering the whole body by stage 26 and joeys achieve a mature appearance by stage 29, the last stage of our study, which culminates with eye opening and weaning at around 70 postnatal days.

## Discussion

Eutherian mammals, such as rodents and humans, differ from marsupials and monotremes in that they have a prolonged intrauterine period that extends beyond embryonic development (past Carnegie/Thieler stage 23). Non-eutherian mammals, on the other hand, are born at comparatively earlier stages and must complete development by feeding from their mothers’ milk, outside the uterus. Accordingly, dunnarts are born with a fully functional mouth apparatus, whereas eutherians at an equivalent stage have unfused pharyngeal arches and orofacial development occurs after the onset of telencephalic development [1, 12, 34]. Another difference is that the anterior skeleton forms much earlier than the posterior skeleton in marsupials, as compared to the more uniform development of eutherians [1, 2, 35–38]. As functional forelimbs are crucial for marsupial embryos to reach the teat, such early behaviour may act as a developmental constraint limiting phenotypic variability of the mouth and forelimbs in marsupials. Accordingly, eutherians display a comparatively larger repertoire of mouth and forelimb morphologies across species than marsupials (e.g. mouth of elephants and star-nosed moles, or forelimbs of bats and dolphins) likely due to the release of such constraints by the extended intrauterine retention of the embryo [34, 39]. Therefore, a major challenge of translating staging systems between species are differences in the relative rates of development between different body parts. However, as the rate of brain development is less variable than that of the body across mammals [11, 40–42], information about the timing of neural structures are optimal for staging purposes. Importantly, later events in brain development are slower in marsupials than in eutherians [7, 10, 11], possibly due to metabolic differences of lactation versus placentation during development [8, 9], further highlighting their potential as animal models brain wiring.

As compared to eutherians, development of the dunnart isocortex is largely protracted and occurs mostly between the second and the sixth postnatal week. The olfactory cortex, on the other hand, acquires a layered organisation much earlier, along with the growth of the lateral olfactory tract and formation of the anterior commissure. It is not clear whether the olfactory and/or vomeronasal systems of dunnarts are functional at birth, however newborn wallabies orient themselves towards odours from the mother’s pouch [43], despite the immature appearance of their olfactory systems [44]. In any case, peripheral and central olfactory structures are present in dunnart newborns, well before the arrival of thalamic inputs from other senses and formation of layers in the isocortex. Whether early olfactory function is related to the subsequent formation of isocortical circuits is a possibility that has ignited recent interest [45–47]. Developmental interactions between the olfactory systems and the rest of the forebrain appear conserved across mammals, even in species whose adults lack functional olfactory systems [48–50]. Moreover, evidence from mice suggest that the olfactory system can influence the cellular and electrical features of isocortical development [51–53], further supporting the hypothesis that the olfactory system could be a driver of mammalian brain development.

The extended period of forebrain formation in marsupials, plus the continuous access to developing joeys inside the pouch, offers promising opportunities to study multiple developmental processes sequentially, and *in vivo*. Such capability cannot be achieved with traditional eutherian models, such as rodents, carnivores and primates, as embryos develop inside the uterus and cannot readily survive if taken prematurely. The excellent breeding under controlled conditions, small body size, large litters, and short life cycles, make dunnarts promising laboratory models to study not only the mechanisms of brain development but also multiple questions of biological relevance.
